# Predictors of Recurrent Urolithiasis in Iran: Findings from a Nationwide Study

**DOI:** 10.34172/aim.2024.29

**Published:** 2024-04-01

**Authors:** Abbas Basiri, Amir Hossein Kashi, Mazyar Zahir, Nasrin Borumandnia, Maryam Taheri, Shabnam Golshan, Behzad Narouie, Hayat Mombeini

**Affiliations:** ^1^Urology and Nephrology Research Center, Shahid Labbafinejad Medical Center, Shahid Beheshti University of Medical Sciences, Tehran, Iran; ^2^Department of Urology, Zahedan University of Medical Sciences, Zahedan, Iran; ^3^Ahvaz Jundishapur University of Medical Sciences, Ahvaz, Iran

**Keywords:** Iran, Recurrence, Risk factors, Urinary calculi, Urolithiasis

## Abstract

**Background:**

Prevention of urinary stone recurrence is the ultimate goal in urolithiasis patients. In this study, we aimed to investigate the national prevalence rate and possible determinants of increased urolithiasis recurrence risk in a nationwide study in Iran.

**Methods:**

All data regarding stone occurrence and recurrence episodes were extracted from the cross-sectional Iran National Stone Survey (INSS) study, and the possible determinants of recurrence were evaluated in the subset of 2913 patients who had a positive history of at least one episode of urolithiasis.

**Results:**

The national prevalence rate of recurrent urolithiasis was 2.6% (95% CI: 2.5, 2.8) in Iran. Moreover, the relative ratio of recurrent stone formers to all stone formers was 39.8% (95% CI: 38.0, 41.6). Our univariable truncated negative binomial regressions suggested that a positive history of urolithiasis in the patient’s father (prevalence ratio [PR] [95% CI]=1.83 [1.39, 2.41], *P*<0.001), mother (PR [95% CI]=1.92 [1.39, 2.66], *P*<0.001) or brother (PR [95% CI]=1.32 [1.03, 1.69], *P*=0.026); and residence in urban areas (PR [95% CI]=1.27 [1.04, 1.55], *P*=0.016) were significant predictors of repetitive recurrence episodes. However, when incorporated into a multivariable truncated negative binomial regression model, the only significant predictors of more frequent recurrence episodes were a positive history in father (PR [95% CI]=1.66 [1.24, 2.22], *P*<0.001) and mother (PR [95% CI]=1.68 [1.20, 2.36], *P*=0.002); and urban residence (PR [95% CI]=1.24 [1.01, 1.51], *P*=0.031).

**Conclusion:**

Our results indicate that a positive family history of urolithiasis in mother and father and residence in urban areas are the significant predictors of recurrence risk in urolithiasis patients in Iran.

## Introduction

 In 2019, urolithiasis was accountable for more than 115 million incident cases and 13 thousand deaths worldwide.^1^ Despite a globally declining trend in age-standardized incidence during the last three decades, some regions (e.g. Middle East, South Asia, and Sub-Saharan Africa) have had substantial increases in this regard.^2^ Moreover, crude incidence rates, disability-adjusted life years (DALYs), and deaths attributed to urolithiasis have increased universally.^1,2^ Specifically, the Middle East and North African region has had an approximately 8% increase in the annual age-standardized incidence rate of urolithiasis.^1^ Moreover, the age-standardized death rate has risen steadily with a mean increase of 1% per year during the last three decades in this region.^2^ These statistics depict the substantial burden imposed by urolithiasis on healthcare systems, especially in the Middle East and North African region.

 Urolithiasis is a complex, multifactorial disease associated with anthropometric status (e.g. obesity), demographic factors (e.g. age and race), genetic predispositions, dietary habits, and external ecologic influencers.^3-6^ The interactions between these risk factors can induce stone formation. For instance, calcium oxalate stones, the most prevalent type of urinary stones, result from the interplay of multiple genes (e.g. claudin gene family, calcitonin receptor gene, calcium sensing receptor gene) and their interaction with dietary (e.g. low calcium, high oxalate) and environmental factors (e.g. long hours of outdoor work and increased perspiration).^7,8^ This interlaced nature of urinary stone development is also observed in other types of urinary stones.^3,5^ Consequently, the multifactorial nature of urolithiasis hinders effective prevention, and although various medical and dietary interventions have been implemented to prevent recurrence, cumulative recurrence rates as high as 50% have been reported.^3,9^

 Our understanding of recurrent urolithiasis is largely based on studies with relatively small sample sizes of previously diagnosed urolithiasis patients or on meta-analyses with various statistical limitations. To the best of our knowledge, there has been no comprehensive nationwide study addressing this issue. In this study, we evaluated the national prevalence rate of recurrent stone formers and the possible contribution of certain risk factors to the recurrence of urolithiasis in a Middle Eastern country.

## Materials and Methods

###  Study Population and Data Source 

 All data regarding stone recurrence were extracted from the Iran National Stone Survey (INSS) study database (National Institute for Medical Research Development [NIMAD] approval number: 989248, ethical review board approval number: IR.NIMAD.REC.1399.113). INSS was a cross sectional national study, conducted from October 2020 to November 2022 in Iran. The protocol of INSS is detailed in a previous manuscript.^10^ In summary, all Iranian nationals who were permanent residents of Iran and had a functional telephone line in the Iranian telecommunications center were included in the study. Exclusion criteria consisted of unwillingness to participate or provide the required information regarding urolithiasis status and failure to verify the data obtained from the participants. Of the total 35,986 residential households who were approached via telephone calls, 11 979 households (33.3%) were successfully contacted and interviewed. They were inquired about the possible occurrence and recurrence of urinary stones among all household members. The mean ± standard deviation number of members in each household who accepted to participate in the study was 4 ± 1, ultimately yielding a total number of 44,186 participants.

 All telephone interviews were made by trained local interviewers to eliminate the potential negative impact of a language barrier. The interviewers were chosen based on their educational level (i.e. a minimum of Bachelor of Science in a health-related field) and dedication. All interviewers completed a comprehensive theoretical program covering scientific aspects (i.e. urolithiasis and its related risk factors) and ethical considerations (i.e. patient confidentiality, informed consent, and data privacy), followed by a briefing session in which the questionnaire was thoroughly discussed with them. The training program was facilitated by the INSS core personnel (i.e. a group of urologists, epidemiologists and biostatisticians). Afterwards, the interviewers directly observed two sample interviews conducted by the INSS core personnel. Lastly, the interviewers were required to conduct two interviews under the direct observation of an INSS core personnel member to ensure their understanding of the questionnaire and familiarity with the required concepts. All interviewers were instructed to seek assistance and scientific support from the INSS core personnel in case they encountered any problems.

 All interviewers were instructed to use the exact same questionnaire, which had been verified and finalized in several meetings of the INSS core personnel. The questionnaire, formatted as a checklist, was prepared based on a thorough literature review and aligned with the objectives of the INSS study. It encompassed various variables related to urolithiasis occurrence and recurrence, including the presence of a urinary stone at the current time, the total number of previous episodes of urolithiasis, family history of kidney stones, demographic determinants, personal history of previous interventions and environmental variables. The checklist was designed to be clear, concise, and comprehensive.

 While a formal validity and reliability test was not performed on the checklist, the INSS core personnel considered the thorough literature review and expert consensus sufficient to ensure its validity and reliability. However, a pilot study was conducted one month prior to the initiation of the original INSS study in order to identify and address any shortcomings of the checklist in practice. The pilot study took place in four provinces with different dialects and varying cultures (i.e. Tehran, Semnan, Ardabil and Sistan-Baluchistan). Ten households were randomly selected and interviewed from each province. The interviews were repeated twice by two different interviewers, and the obtained information was evaluated by an external observer with regard to agreement, possible errors, inconsistencies, ambiguities, and missing data. The respondents were also asked to provide their feedback on the clarity of the questions. Based on the results of the pilot study, the checklist was then revised and improved before the main study.

 In terms of data quality control, all data collected by the interviewers were double-checked by a designated member of the INSS core personnel. If any of the questions were left unanswered, the interviewer was instructed to call that participant again and repeat the specific question. In case any of the questions remained unanswered during the second interview, it was then considered as missing data. Additionally, data concerning family members who could not be reached or verified were also excluded from the INSS database.

 The phone numbers and medical data of all participants remained confidential during and after the study and all the interviewees were informed of their right to withdraw their data until three months after the interview and before the data were entered into the finalized data pool. Moreover, if any patient had any specific questions regarding their kidney stone disease, they were contacted again by the INSS core personnel to counsel them on their disease and possible required treatments.

 Lifetime prevalence of recurrent urolithiasis was defined as a self-reported history of at least two urinary stones episodes, detected by either imaging or spontaneous stone passing. The lifetime prevalence of recurrent urolithiasis was calculated as the percentage of participants with ≥ 2 urinary stones episodes out of the total number of participants. The unit of measurement was per person. Similarly, the ratio of recurrent stone formers to all stone formers was determined by dividing the total number of participants with ≥ 2 urinary stones episodes by all patients with at least one episode of urinary stone disease, measured per person and expressed as a percentage. To explore the contribution of demographic, personal and familial risk factors to recurrence, data were extracted and analyzed from the subset of 2913 patients who had reported at least one episode of urolithiasis during their lifetime. Patients’ follow-up times were calculated by subtracting the age at the time of the first stone episode from the age at the time of the study. The total episodes of urinary stone recurrence during this follow-up times were then used to build respective predictive models.

###  Statistical Analysis 

 All data were analyzed using SPSS version 23 (IBM, Armonk, NY, United States) and Stata version 14 (StataCorp LLC, TX, United States). The data were described as frequency (percentage) for qualitative variables, and as mean ± SD or median (interquartile range) for quantitative variables. Univariable truncated negative binomial regressions were used to calculate the prevalence ratios (PRs) of potential risk factors. PRs were actually the calculated exponentiated coefficients (Exp(β)) used to assess the relative strength of association between risk factors and the number of recurrence episodes. Considering the cross-sectional design of our study, these PRs provided valuable insights into the relationship between risk factors and recurrence risk. Truncated negative binomial regression is used for analyzing over-dispersed count data that have a lower bound greater than zero. This implies that the response variable (total episodes of urinary stone recurrence) is constrained from assuming the value of zero, and solely constructive counts can be observed.^11^ In other words, this statistical model is used to model ‘count data’ that are all above a certain value, called the truncation point. In our study, we only included patients who had a positive history of at least one episode of urolithiasis, so our data were truncated at zero. To account for the different follow-up times in samples, the ‘patients’ follow-up times’ were incorporated into the model as an exposure variable. This variable indicates the amount of time each patient was at risk of having a stone recurrence, and the expected number of episodes was proportional to this time. Therefore, the results were adjusted for the patients’ follow-up times because patients who had a longer follow-up time were more likely to experience another stone episode. A predictive multivariable model was then fitted by utilizing multivariable truncated negative binomial regression. In all statistical analyses, a *P* value < 0.05 was considered statistically significant.

## Results

###  Recurrence, Demographic Characteristics and Geographical Distribution 

**Figure 1:**
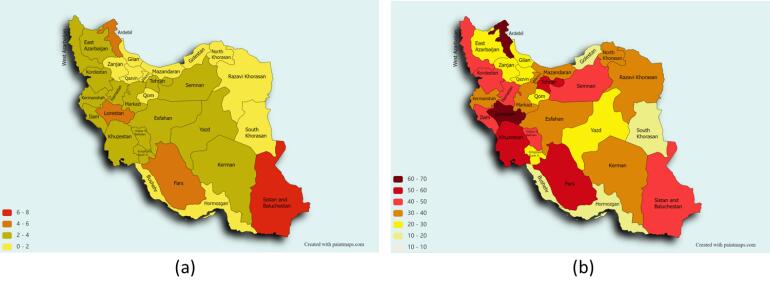
Geographic Heat Maps of (a) the Lifetime Prevalence Rate of Recurrent Stone Formers and (b) the Ratio of Recurrent Stone Formers to All Stone Formers, Both measured per person and expressed as percentages

 Our patient population comprised of 1741 (59.8%) men and 1172 (40.2%) women. The mean age of our patient population was 49.2 ± 15.9 years. A total of 609/2547 (23.9%) participants reported previous extracorporeal shock wave lithotripsy, while 315/2551 (12.3%) participants had previous surgical intervention. Table 1 depicts the descriptive characteristics of our patient population stratified based on urinary stone recurrence. As observed, 1159 recurrent stone formers were identified in total. Considering the total population of the study (N = 44186), this translates into a 2.6% [95% CI: 2.5, 2.8] lifetime prevalence of recurrent stone formers in Iran. As illustrated in Figure 1a, the highest and lowest life time prevalence rates of recurrent stone formers were seen in the southeastern (Sistan-Baluchistan province; 6.8% [95% CI: 5.6, 8.1]) and northern (Golestan province; 0.4% [95% CI: 0.1, 0.9]) regions of Iran, respectively.

**Table 1:** Possible Contributors to Urolithiasis Recurrence.

**Variable**			**Urinary stone episodes**		**Univariable models**		**Multivariable model **	
			**One ( N=1754)**	**>One (recurrent)** **(N=1159)**	Prevalence ratio* **(95% CI)**	***P***	Prevalence ratio* ** (95% CI)**	***P***
Follow-up time (year)			5.0 [IQR: 2.0, 10.0]	8.0 [IQR: 4.0, 15.0]	*Incorporated in the model as an exposure variable*			
Gender	Female		730 (41.6%)	442 (38.1%)	Reference		Reference	
	Male		1024 (58.4%)	717 (61.9%)	0.91 (0.76, 1.09)	0.296	0.93 (0.78, 1.11)	0.463
Age at first urolithiasis episode (year)^**^			41.8 ± 14.9	39.4 ± 13.7	1.00 (0.99, 1.00)	0.278	1.00 (0.99,1.01)	0.260
Positive family history	Father	No	1608 (91.7%)	999 (86.2%)	Reference		Reference	
		Yes	146 (8.3%)	160 (13.8%)	1.83 (1.39, 2.41)	< 0.001	1.66 (1.24, 2.22)	0.001
	Mother	No	1642 (93.6%)	1050 (90.6%)	Reference		Reference	
		Yes	112 (6.4%)	109 (9.4%)	1.92 (1.39, 2.66)	< 0.001	1.68 (1.20, 2.36)	0.002
	Sister	No	1633 (93.1%)	1023 (88.3%)	Reference		Reference	
		Yes	121 (6.9%)	136 (11.7%)	1.24 (0.92, 1.65)	0.147	1.07 (0.79, 1.46)	0.641
	Brother	No	1582 (90.2%)	950 (82.0%)	Reference		Reference	
		Yes	172 (9.8%)	209 (18.0%)	1.32 (1.03, 1.69)	0.026	1.16 (0.89, 1.51)	0.250
Urbanization status	Rural		512 (29.2%)	285 (24.6%)	Reference		Reference	
	Urban		1242 (70.8%)	874 (75.4%)	1.27 (1.04, 1.55)	0.016	1.24 (1.01, 1.51)	0.031

All data are expressed as count (percentage), mean ± SD or median [IQR]. ^*^PRs were exponentiated coefficients (Exp(β)) estimated by truncated negative binomial regression models after adjusting for the differences in follow-up time by incorporating follow-up time as an exposure variable in the model. ^**^In the case of age, PR was estimated as the relative risk of developing recurrent urolithiasis episodes per each decade of increase in age at the time of the first urinary stone episode.

 Moreover, considering the 1159 recurrent stone formers among the total 2913 urolithiasis patients who had at least one urinary stone episode during their lifetime, the ratio of recurrent stone formers to all stone formers was calculated to be 39.8% [95% CI: 38.0, 41.6]. Regarding geographical distribution, the ratio of recurrent stone formers was highest in the northwestern province of Ardebil (63.0% [95% CI: 49.7, 74.9]) and lowest in the southern province of Bushehr (14.0% [95% CI: 6.9, 24.7]). Figure 1b depicts the geographic heat map of the ratio of recurrent stone formers to all stone formers in Iran.

 Given that our data on age at stone episodes was limited to the age of the patients at their first stone episodes and their age at the time of the study, we utilized data from patients who had only one episode of recurrence, which occurred simultaneously with the study’s conductance date (N = 89). This allowed us to calculate an estimated median time to recurrence. The estimated median time to recurrence was 4.0 (IQR: 2.0, 10.0) years.

###  Determinants of Stone Recurrence 

 As demonstrated in Table 1, our univariable analyses revealed that a history of urolithiasis in the patient’s father (*P* < 0.001), mother (*P* < 0.001) or brother (*P* = 0.026) was associated with an increased risk of recurrent urinary stone events; but a positive history in the patient’s sister was not associated with an increased risk of stone recurrence (*P* = 0.147). Moreover, residence in urban areas (*P* = 0.016) had a significant effect on the risk of stone recurrence. Nevertheless, age at first urolithiasis episode and sex were not significant contributors to increased urolithiasis recurrence risk (*P* = 0.278 and *P* = 0.296, respectively). We then incorporated all the variables into a multivariable model (Table 1). This multivariable analysis revealed that a positive history in the patient’s father (*P* = 0.001) and mother (*P* = 0.002) and residence in urban areas (*P* = 0.031) were significant predictors of recurrent urolithiasis. However, a positive history in the patient’s brother (*P* = 0.250) and sister (*P* = 0.641), sex (*P* = 0.463) and age at first urolithiasis episode (*P* = 0.260) were not significantly associated with urinary stone recurrence according to the multivariable analysis.

## Discussion

 The primary concern of almost every urolithiasis patient is to prevent another agonizing episode of urinary stone event. Historically, studies suggest a 50% risk of recurrence at 10 years.^9,12^ According to our findings, the lifetime prevalence rate of recurrent stone formers was 2.6% in Iran and the approximate median time to first recurrence was 4.0 (IQR: 2.0, 10.0) years. Our estimated time to first recurrence was meaningfully higher than a previous report from Iran, which reported a median time to recurrence of 21 months in 2007.^13^ Although the sample size we used to determine the median time to first recurrence was relatively smaller than the aforementioned study, our study design appears to be more robust, making our findings more reliable in this regard. Moreover, other international studies on both adult and pediatric urolithiasis patients, have reported relatively similar results to ours, with the time to initial recurrence ranging from three to five years.^14-16^

 The significant role of family history in urinary stone recurrence has been established previously and even integrated into predictive nomograms.^3,17-19^ Nonetheless, to the best of our knowledge, there has been no study on the possible differential influence that positive family history in different family members can have on repetitive urolithiasis recurrence episodes. Our multivariable analysis revealed that a positive history in the patient’s mother or father was adequate for predicting the risk of recurrence episodes among patients. Contrary to the previous studies that have demonstrated a significant role for positive history in the brother or sister in increasing the risk of first urolithiasis recurrence, our model did not support these findings.^18,19^

 Furthermore, our multivariable model did not support the possible role of patient’s age at the first stone episode or patient’s sex in altering the risk of urinary stone recurrence. Previously, some studies suggested that younger age at the first urinary stone episode may expedite the first stone recurrence event.^18,19^ Although they did not evaluate the underlying scientific rationale for this finding, it appeared that younger age at first presentation may be an indicator of higher genetic susceptibility to recurrent urinary stone disease. Nevertheless, our national level data challenged this notion, demonstrating that age at the time of the first urolithiasis episode, is not associated with the probability of more frequent recurrences in the rest of a patient’s life. Former studies have failed to reach consensus on the possible contribution of patient’s sex to urinary stone recurrence, with most studies demonstrating male sex as a major contributor to recurrence,^18,19^ while others propose female sex as a significant risk factor.^20^ Our data failed to contribute to this scientific discourse and only suggested that patient’s sex appears to be unrelated to stone recurrence.

 An interesting finding of our study was the detrimental effect of urbanization in increasing the risk of urinary stone recurrence episodes. In parallel, previous studies have suggested that urbanization can accentuate the risk of developing urinary stones.^21^ However, as argued by Goldfarb and Hirsch, considering the simultaneous confounding effect of urbanization on patients’ diet, occupation, and income, estimation of its net effect on urinary stone disease and recurrence is difficult, if not impossible.^22^ Conclusively, it is only hypothesized that urbanization may increase the risk of consecutive urinary stone recurrence events.

 With regards to geographical distribution, our analyses revealed that the life-time prevalence of recurrent urolithiasis events was highest in the southeastern province of Sistan-Baluchistan and lowest in the northern province of Golestan. These findings were predictable according to the original INSS study which demonstrated a relatively similar distribution of the lifetime prevalence of urolithiasis, regardless of the number of urinary stone episodes.^10^ Nevertheless, the ratio of recurrent stone formers to all stone formers was highest in the northwestern province of Ardebil but lowest in the southern province of Bushehr. It is worth mentioning that a recent study has shown that urbanization is fairly higher in the western and northwestern regions of Iran in comparison with the southern and southeastern provinces.^23^ Considering our results that suggested a significant role for urbanization in susceptibility to more frequent recurrence episodes, it is deducible that this high frequency of recurrent stone formers relative to all stone formers may be due to this factor. Moreover, a previous study has delineated the genetic differences among various Persian ethnicities,^24^ which may be another possible explanation for the observed differences between different provinces, each of which are mostly inhabited by certain ethnic groups.

 Our study was subject to some of the inherent limitations and biases of all interview-based studies. A possible source of bias was recall bias, as the INSS study relied on the self-reported history of urolithiasis and recurrence among the participants; some participants may have forgotten or misreported the number or timing of their stone episodes, which could affect the accuracy of our analyses. A second potential source of bias in the INSS study was proxy bias, which occurs when the data are collected from a person who is not the actual subject of the study. In the INSS study, the people who answered the phone calls were asked to give information about household members other than themselves. This may have introduced misreporting, depending on the relationship and awareness of the respondent to the family members’ health status. Nevertheless, the interviewers were instructed to confirm the information with the family members whenever possible in order to minimize this source of bias. Moreover, as mentioned above, data of the family members who could not be reached or verified were excluded from the INSS database. Another possible source of bias was selection bias, as the INSS study was conducted using telephone interviews to collect the data. As observed, only 33.3% answered the phone calls and were willing to participate in the study. Moreover, the regional disparity in access to telephone lines might have further affected the findings. Nevertheless, the INSS study tried to address this bias by making phone calls proportionate to the ratio of each province population to the national population. A fourth possible source of bias was information bias. Some questions may have been unclear or ambiguous for the participants or they might have refused to answer some questions. However, the INSS core personnel tried to minimize this bias by conducting a pilot study, omitting vague and controversial questions and most importantly, employing local telephone interviewers (especially in Sistan-Baluchistan, Bushehr, Khuzestan and Turkish-speaking provinces), who were adequately trained and closely familiar with the language and culture of the people of each region. Lastly, some of the possible determinants of recurrence were inevitably eliminated due to the limited time of each interview. Despite all these limitations, the nationwide design of the study and having the largest sample size of urolithiasis patients ever reported in Iran make our findings ponderable.

## Conclusion

 In conclusion, our study showed that the national lifetime prevalence of recurrent urolithiasis episodes and the relative ratio of recurrent stone formers were 2.6% and 39.8%, respectively. According to our findings, positive history of urolithiasis in the patient’s father and mother and residence in urban regions appear to play a significant role in increasing the risk of experiencing urolithiasis recurrence.
